# The ultrasound-based cardiac output monitoring is a useful tool to define baseline hemodynamic parameters in healthy permanent residents at high altitude: results of a monocentric pilot study

**DOI:** 10.3389/fphys.2023.1297872

**Published:** 2024-01-17

**Authors:** Antonio Viruez-Soto, Daniel Molano-Franco, Alfredo Merino-Luna, Aida Bairam, Fernanda Aliaga-Raduán, Lida Sanchez, Christian Arias-Reyes, Jorge Soliz

**Affiliations:** ^1^ High Altitude Intensive Care Medicine International Group, GIMIA, La Paz, Bolivia; ^2^ High Altitude Intensive Care Medicine International Group, GIMIA, Bogota, Colombia; ^3^ Carrera de Medicina Humana, Facultad de Ciencias de la Salud, Universidad San Ignacio de Loyola, Lima-Perú, Peru; ^4^ Centre de Recherche de l’Institute Universitaire de Cardiologie et de Pneumologie de Québec, Université Laval, Québec, QC, Canada; ^5^ Bolivian Foundation of Altitude Sciences (BFAS), Brain Research Institute, La Paz, Bolivia

**Keywords:** hypobaric hypoxia, cardiac output, hemodynamic, ultrasound, sex dimorphism

## Abstract

Previous studies on the cardiac data of healthy permanent residents living in high-altitude regions such as Tibet and the Andes have yielded inconsistent findings and significant disparities. These discrepancies can be mainly attributed to the invasive methods conventionally used for parameter evaluation. However, with the introduction of cutting-edge ultrasound technology, there is now an innovative approach to addressing and reconciling these variations. In this pilot study, we employed an ultrasound-based cardiac output monitoring (USCOM) device to evaluate cardiac output and related hemodynamic variables in a group of 20 healthy high-altitude Andean residents (comprising 10 men and 10 women) aged between 26 and 35 years old. The monocentric study was carried out in La Paz, Bolivia, located between at an altitude of 3,600–4,000 m. A total of 60 hemodynamic measurements were evaluated, accounting for three technical replicates per subject. Our results showed strong intrasubject reproducibility and revealed important differences related to both sex and hemodynamic parameters in highlanders compared to individuals residing at sea level. We conclude that USCOM represents a highly reliable technology for performing hemodynamic measurements in high-altitude residents. Our preliminary findings underscore the need for larger studies, encompassing larger sample sizes, specifically tailored to gender considerations, and extendable to broader highland populations. These findings have special significant implications for the management of hemodynamics in intensive care and postoperative settings, warranting further comprehensive research efforts.

## Introduction

Pulmonary artery catheterization is considered the conventional technique for measuring cardiac output by thermodilution in humans (Harvey et al., 2005; van Lelyveld-Haas et al., 2008). However, it is accompanied by substantial controversies, primarily stemming from the inherent risks involved. These risks encompass life-threatening complications, such as pulmonary infarction and ventricular free wall rupture (Ivanov et al., 2000; Rhodes et al., 2002), the imperative need for confirming an optimal catheter position, the requisite training for proficient catheter implantation, and the complexities associated with data interpretation (Dey and Sprivulis, 2005; van Lelyveld-Haas et al., 2008). These disadvantages make the application of the procedure in research highly challenging. Conversely, there is an urging demand for the non-invasive, reproducible, and repeatable estimation of cardiac output at the bedside, particularly in the care of hemodynamically unstable patients (Rivers et al., 2001; van Lelyveld-Haas et al., 2008). In response to this clinical need, the ultrasonic cardiac output monitor (USCOM) has emerged as a valuable alternative. Utilizing continuous wave Doppler ultrasound with frequencies ranging from 2.0 to 3.3 MHz, USCOM delivers real-time non-invasive transcutaneous cardiac output measurements. Developed in 2011 and introduced for clinical use in the Netherlands in 2015, this technology has rapidly gained international acclaim for its ease of use, even by personnel lacking prior ultrasound training (Dey and Sprivulis, 2005; Tan et al., 2005; van Lelyveld-Haas et al., 2008).

However, all studies validating the clinical applicability of USCOM have thus far been conducted exclusively in patients at sea level. No investigations have ventured into the utilization of USCOM technology among individuals residing permanently at high altitudes (2,500–3,600 masl) and very high altitudes (3,600–5,500 masl) (Viruez-Soto et al., 2022). Given that over 150 million individuals permanently inhabit areas above 2,500 m above sea level worldwide, the paucity of knowledge regarding the hemodynamics of this population is of paramount significance (Joseph et al., 2000; Zubieta-Calleja et al., 2011). Previous research on this topic is limited to old studies, revealing considerable variation in cardiac index (cardiac output normalized by body surface area, accounting for body size) in highlanders. This variability is likely due to the challenges associated with the catheterization technique, as outlined in previous reviews (Bell and Strohl, 2021). In light of these considerations, the primary objective of this pilot study is to validate the utility of USCOM technology in elucidating the hemodynamic profiles of healthy Andean permanent residents of La Paz, Bolivia (3,600–4,000 masl).

## Methods

### Ethics declaration

The study was approved by the Bioethics Institutional Committee of the “Clínica Los Andes del Grupo Embriovid” in La Paz, Bolivia, and was carried out in accordance with the Helsinki Declaration. All participants signed and gave informed consent.

### Study design

We conducted an observational, descriptive cross-sectional study at the “Clínica Los Andes del Grupo Embriovid” in the city of La Paz, Bolivia. The study was carried out during the November 2022—April 2023 period. The study sample was taken from a group of people that were easy to contact and reach.

### Inclusion and exclusion criteria

Study subjects were included if they were: 1) healthy volunteers, 2) between 25 and 45 years old, 3) permanent high-altitude residents from La Paz (between 3,600 and 4,000 m above sea level), with no history of migration from the lowlands in the past year. Subjects with one or more of the following criteria were not considered: 1) people that refused to provide informed consent, and 2) people with a history of diagnosis or treatment of heart disease, lung disease, blood cancer, or chronic mountain sickness (CMS).

### USCOM technology

The intricacies of this apparatus have been detailed in other references (Cattermole et al., 2017; Vinayagam et al., 2018) and the instrument’s user manual (http://site.uscom.com.au/dist/train/education/The_USCOM_and_Haemodynamics.pdf). In essence, ultrasonic cardiac output monitor-1A (USCOM-1A; Pty Ltd., Coffs Harbour, NSW, Australia) is a direct branch of echocardiography, employing continuous wave Doppler ultrasound for meticulous measurement of hemodynamic indices. In pursuit of precision, USCOM uses the suprasternal insonation window for the aortic valve or the left parasternal window for the pulmonary valve, thereby extracting the velocity-time integral (VTI) of ejection flow and heart rate (HR). A distinctive algorithm, depending on height (for subjects over 50 cm) or weight (less than 50 kg), is used to extrapolate the cross-sectional area (CSA) of the two valves. Stroke volume is materialized by the equation SV = CSA × VTI. Heart rate is distinguished from the systolic interjection interval, simultaneously taking into account systolic and diastolic blood flows. Manual entry of pressure values (systolic, SBP; diastolic, DBP) sets the stage for the calculation of mean arterial pressure (MAP) using MAP = DBP + ([SBP - DBP]/3). Finally, USCOM undertakes the calculation of the body surface area (BSA) using the traditional formula of Du Bois and Du Bois (1916). From this basis, the following parameters were recorded or calculated: BSA-indexed values for cardiac output (CO), cardiac index (CI), systemic vascular resistance (SVR), systemic vascular resistance index (SVRI), stroke volume (SV), stroke volume index (SVI), oxygen delivery (DO_2_), oxygen delivery index (DO_2_I), peak velocity (Vpk), and stroke volume variation (SVV).

Mean arterial pressure (MAP) and oxyhemoglobin saturation (SatpO_2_) in room air were monitored using pulse oximetry (Dräger Infinity M540 Monitor, Germany). The average hemoglobin value for high altitude (men:16.8 g/dL; women: 14.5 g/dL) (Viruez-Soto et al., 2020), and an average central venous pressure of 6 mmHg (for men and women) (Shah and Louis, 2023) were used in the USCOM software.

## Evaluation protocol

In the evaluation room, participants were given 5 min to rest in the supine position. Personal identification and contact data were then recorded, along with data corresponding to sex, age, and weight. All information was duly anonymized and encrypted. The hemodynamic profile was obtained using a 2.2 MHz transducer placed on the suprasternal window with aortic focus. A single operator, previously trained to use the USCOM-1A system, collected all data for this study. Three consecutive diagnostic-quality Doppler ejection profiles were performed for each measurement, and three measurements were performed for each subject. The values obtained were compared with the reference values established by the manufacturer for the USCOM-1A, according to age group.

### Sample size calculation

The sample size required for the detection of statistically significant effects indicative of disparities between the reference means and the calculated means for cardiac output in male and female patients, was calculated by using the conventional formula:

$$N=\frac{\sigma^{2}{\left(z1-\beta +z1-\frac{\alpha }{2}\right)}^{2}}{{\left(\mu 0-\mu 1\right)}^{2}}$$

Where:

*μ*_0_ = reference mean *μ*_1_ = mean of age group (per sex) N = required sample size of study population (age group - per sex) σ = variance of age group (per sex) α = probability of type I error (usually 0.05) β = probability of type II error (usually 0.2) z = critical Z value for a given α or β

Considering the unavailability of means in the reference intervals provided by the USCOM manufacturer, we derived the reference means by identifying the central value within the reference interval and approximated the standard deviation to one-quarter of the range spanned by the reference interval. For these calculations we maintained a significance level (α) of 0.05 and a statistical power of 80%. The sample sizes required to detect a 10% drop for each USCOM parameter are detailed in Table 1.

**TABLE 1:** Sample sizes required to detect a 10% drop in USCOM parameters.

Parameter	Reference mean ± S.D.(provided by the manufacturer)	Required sample size
CO (L/min)	5.8 ± 0.5	6
CI (L/min/m^2^)	3.55 ± 0.325	6
SVR (din/s/cm^-5^)	1215.5 ± 183.75	57
SVRI (din/s/cm-5/m^2^)	2110.5 ± 328.25	19
SV (mL)	76 ± 6.5	5
SVI (mL/m^2^)	42.5 ± 3.75	6
DO2 (mL/min)	1105 ± 97	6
DO2I (mL/min/m^2^)	664.5 ± 59.25	6
Vpk (m/s)	1.2 ± 0.1	5
SVV (%)	21 ± 4.5	40

### Statistical analysis

All statistical tests were performed in IBM SPSS Statistics for Windows, Version 26.0. (Armonk, NY: IBM Corp. IBM Corp. Released 2019) unless stated otherwise.

USCOM parameters: CO, CI, SVR, SVRI, SV, SVI, DO_2_, DO_2_I, Vpk, and SVV were categorized according to sex. The effect of sex on the measured USCOM parameters was tested by a two-way repeated measures MANOVA where CO, CI, SVR, SV, DO_2_, Vpk, and SVV were the dependent variables. Sex was the independent variable, while the three measurements taken from each patient for each USCOM parameter were the repeated measures. Test-retest reliability was assessed by intraclass coefficient analysis using a two-factor mixed-effects model (type: single measurements, definition: absolute agreement).

The sex-specific reference intervals for high-altitude individuals were estimated as the 2.5 and 97.5 percentiles using the robust method according to the guidelines of the CLSI (C28-A3) from the average of the three repeated measures on each subject, using MedCalc^®^ Statistical Software version 20.217 (MedCalc Software Ltd., Ostend, Belgium; https://www.medcalc.org; 2023). The sex-specific reference intervals for each USCOM parameter in high-altitude individuals were compared to those of sea-level individuals reported by the manufacturer (CO: 4.8–6.8; CI: 2.9–4.2; SVR: 848–1583; SVRI: 1454–2767; SV: 63–89; SVI: 35–50; DO_2_: 911–1299; DO_2_I: 546–783; Vpk: 1–1.4; SVV: 12–30; https://www.talentmed.com.tw/download/UscomManual_English.pdf) using Moses extreme reactions tests.

## Results

### Readings with USCOM in high altitude residents show great reproducibility

In this study, a total of 60 measurements (three technical replicates per individual) of the hemodynamic profile were performed on 10 healthy female volunteers and 10 healthy male volunteers between 26 and 35 years old. All participants were habitual La Paz residents, located between 3,600 and 4,000 masl. None had a migratory history, at least during the previous year. Data on sex, height, weight, as well as baseline hemodynamic profile, are described in Table 2. A robust test re-test reliability in USCOM readings was evidenced (interclass correlation = 0.9, CI_95%_: 0.56–0.88).

**TABLE 2:** Description of the studied sample. Values of Age, Height, Weight, BSA (Body surface area), MAP (Mean arterial pressure), SatpO_2_ (Oxygen saturation), HR (Heart rate), MD (mean distance), CO (cardiac output), CI (cardiac index), SVR (systemic vascular resistance), SVRI (systemic vascular resistance index), SV (stroke volume), SVI (stroke volume index), DO_2_ (arterial oxygen delivery), DO_2_I (arterial oxygen delivery index), Vpk (peak ejection velocity), and SVV (stroke volume variation) are shown as mean ± SD.

	Female (*n* = 10)	Male (*n* = 10)
**Age (years)**	31.7 ± 2.11	31.7 ± 2.79
**Height (cm)**	158 ± 6.15	173.3 ± 4.27
**Weight (Kg)**	60.2 ± 9.38	78.1 ± 8.16
**BSA (m** ^**2**^ **)**	1.62 ± 0.13	1.94 ± 0.12
**MAP (mmHg)**	78.72 ± 6.23	85.2 ± 5.22
**SatpO**_**2**_ **(%)**	91.14 ± 1.41	91.33 ± 0.93
**HR (bpm)**	64.5 ± 8.41	69.78 ± 8.65
**MD (m/min)**	15.86 ± 1.23	16.1 ± 0.49
**CO (L/min)**	4.04 ± 0.58	4.91 ± 0.37
**CI (L/min/m** ^**2**^ **)**	2.5 ± 0.34	2.53 ± 0.17
**SVR (din/s/cm** ^**-5**^ **)**	1221.33 ± 203.94	1249.9 ± 183.47
**SVRI (din/s/cm** ^**-5**^ **/m** ^**2**^ **)**	1982.57 ± 377.05	2436.97 ± 462.84
**SV (mL)**	62.7 ± 3.69	70.73 ± 4.36
**SVI (mL/m** ^**2**^ **)**	39 ± 3.28	36.8 ± 3.05
**DO**_**2**_ **(mL/min)**	728.4 ± 112.7	1023.63 ± 77.42
**DO**_**2**_ **I (mL/min/m** ^**2**^ **)**	449.67 ± 65.24	528.37 ± 35.66
**Vpk (m/s)**	1.13 ± 0.07	1.17 ± 0.05
**SVV (%)**	22.17 ± 5.63	17.13 ± 1.15

### USCOM readings reveal sex-related differences in hemodynamic profile in high altitude residents

**FIGURE 1:**
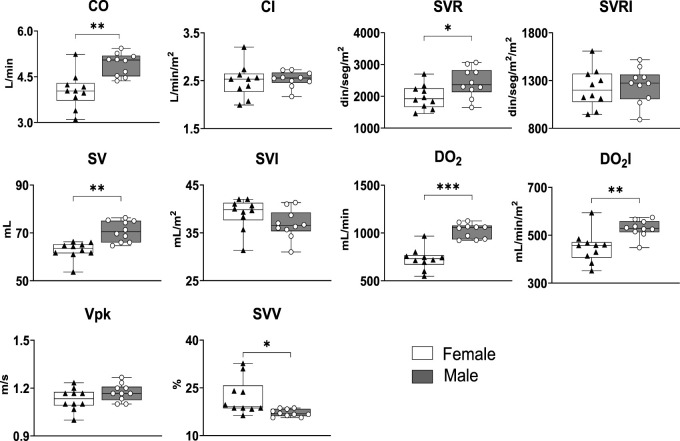
USCOM parameters are sex dependent in high altitude residents. Whiskers represent minimum and maximum values. Horizontal bars within the boxes show the median. *: *p* < 0.05; **: *p* < 0.01; ***: *p* < 0.001. *n* = 10.

Sex-related differences in the cardiorespiratory physiology of highlanders are frequently observed. Thus, we explored potential sex-related differences in the hemodynamic parameters of highlanders measured by USCOM. Two-way MANOVA analyses performed unveiled lower values of CO (*p* = 0.001), SVR (*p* = 0.017), SV (*p* = 0.001), DO_2_ (*p* < 0.0001), DO_2_I (*p* = 0.002), and SVV (*p* = 0.017) in female than in their male counterparts. (*p* = 0.016—Table 2 and Figure 1).

### Establishing New Hemodynamic Reference Intervals for High Altitude Residents Based on USCOM Measurements

**FIGURE 2:**
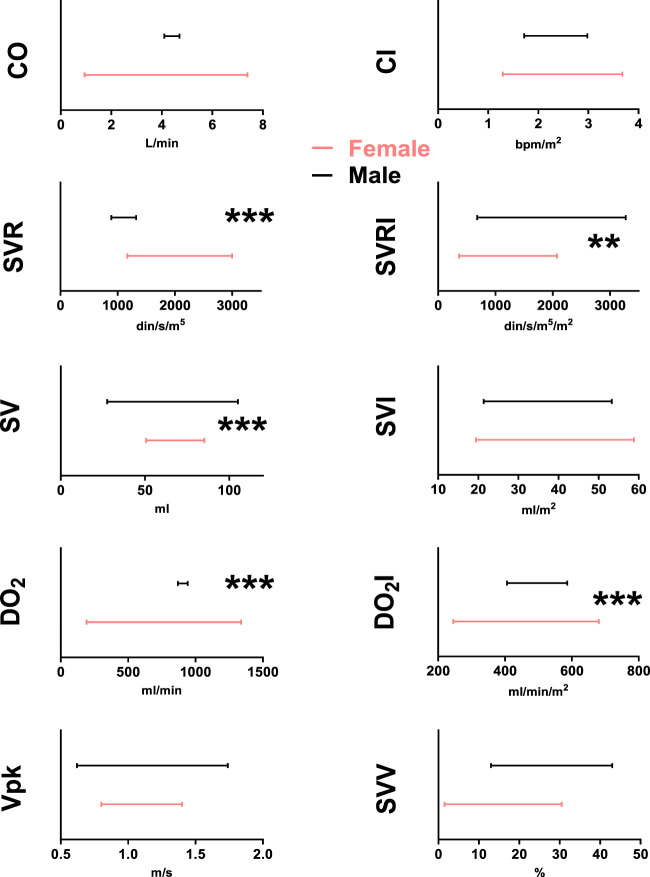
USCOM reference intervals for healthy male adult (black lines) and female (red lines) high-altitude residents for CO (cardiac output), CI (cardiac index), SVR (systemic vascular resistance), SVRI (systemic vascular resistance index), SV (stroke volume), SVI (stroke volume index), DO_2_ (arterial oxygen delivery), DO_2_I (arterial oxygen delivery index), Vpk (peak ejection velocity), and SVV (stroke volume variation). ***: *p* < 0.001 male vs. female.

From the distribution of the values obtained from the corresponding hemodynamic variables grouped by sex, the reference intervals for healthy high-altitude individuals were calculated. For this, the 2.5 and 97.5 percentiles were used as recommended in the CLSI guidelines (C28-A3) and the statistical software MedCalc^®^ was used (Table 3). Next, specific reference intervals were compared between high altitude men and women using Moses’ extreme reaction tests. In accordance with the previously calculated results indicating sexual dimorphism (Figure 2), the comparison by sex showed that the new reference intervals for high-altitude women are significantly different from those of high-altitude men in SVR, SVRI, SV, DO_2_, DO_2_I (Table 4). These results reveal that the hemodynamic parameters of permanent high-altitude residents are dependent on sex.

**TABLE 3:** Reference intervals for high-altitude residents.

	Female (*n* = 10)	Male (*n* = 10)
**CO (L/min)**	3.02–5.89	2.69–6.39
**CI (L/min/m** ^**2**^ **)**	2.08–2.96	1.75–3.4
**SVR (din/s/cm** ^**−5**^ **)**	950.5–3600.78	694.54–1540.54
**SVRI (din/s/cm** ^**-5**^ **/m** ^**2**^ **)**	792.35–1793.49	887.29–2906.05
**SV (mL)**	55.44–78.67	47.82–83.97
**SVI (mL/m** ^**2**^ **)**	29.7–52.85	34.19–42.3
**DO**_**2**_ **(mL/min)**	455.65–1303.23	399.05–1368.37
**DO**_**2**_ **I (mL/min/m** ^**2**^ **)**	354.39–621.16	314.67–685.47
**Vpk (m/s)**	0.91–1.38	1–1.3
**SVV (%)**	13–43	1.51–30.49

CO (cardiac output), CI(cardiac index), SVR (systemic vascular resistance), SVRI (systemic vascular resistance index), SV (stroke volume), SVI (stroke volume index), DO_2_ (arterial oxygen delivery), DO_2_I (arterial oxygen delivery index), Vpk (peak ejection velocity), and SVV (stroke volume variation) are shown as mean ± SD.

**TABLE 4:** Moses extreme reactions test results for the comparison between HA women’s and men’s reference intervals.

**CO (L/min)**	G* = 16, *p* = 8.48
**CI (L/min/m** ^**2**^ **)**	G* = 18, *p* = 1
**SVR (din/s/cm** ^**−5**^ **)**	G* = 8, *p* < 0.001
**SVRI (din/s/cm** ^**−5**^ **/m** ^**2**^ **)**	G* = 9, *p* = 0.01
**SV (mL)**	G* = 17, *p* = 0.957
**SVI (mL/m** ^**2**^ **)**	G* = 11, *p* = 0.089
**DO**_**2**_ **(mL/min)**	G* = 15, *p* = 0.686
**DO**_**2**_ **I (mL/min/m** ^**2**^ **)**	G* = 16, *p* = 0.848
**Vpk (m/s)**	G* = 15, *p* = 0.686
**SVV (%)**	G* = 14, *p* = 0.5

CO (cardiac output), CI(cardiac index), SVR (systemic vascular resistance), SVRI (systemic vascular resistance index), SV (stroke volume), SVI (stroke volume index), DO_2_ (arterial oxygen delivery), DO_2_I (arterial oxygen delivery index), Vpk (peak ejection velocity), and SVV (stroke volume variation) are shown as mean ± SD.

### The reference intervals for hemodynamic parameters in permanent high-altitude residents exhibit significant differences from those of individuals residing at sea level

**FIGURE 3:**
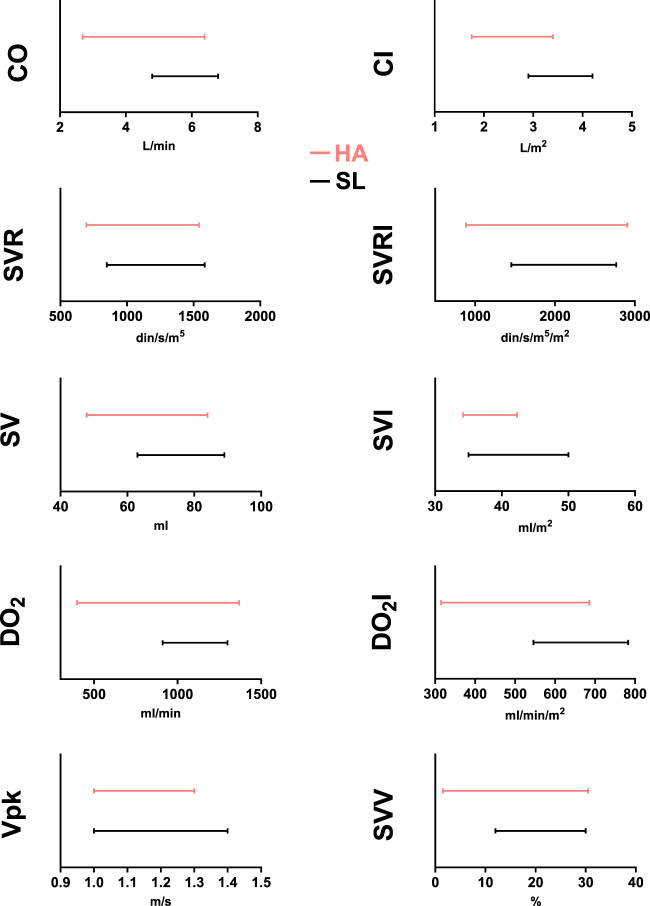
USCOM reference intervals for healthy male adult high-altitude (3,600 m–red lines) and sea-level (black lines) residents for CO (cardiac output), CI (cardiac index), SVR (systemic vascular resistance), SVRI (systemic vascular resistance index), SV (stroke volume), SVI (stroke volume index), DO_2_ (arterial oxygen delivery), DO_2_I (arterial oxygen delivery index), Vpk (peak ejection velocity), and SVV (stroke volume variation). ***: *p* < 0.001 HA vs. SL.

**FIGURE 4:**
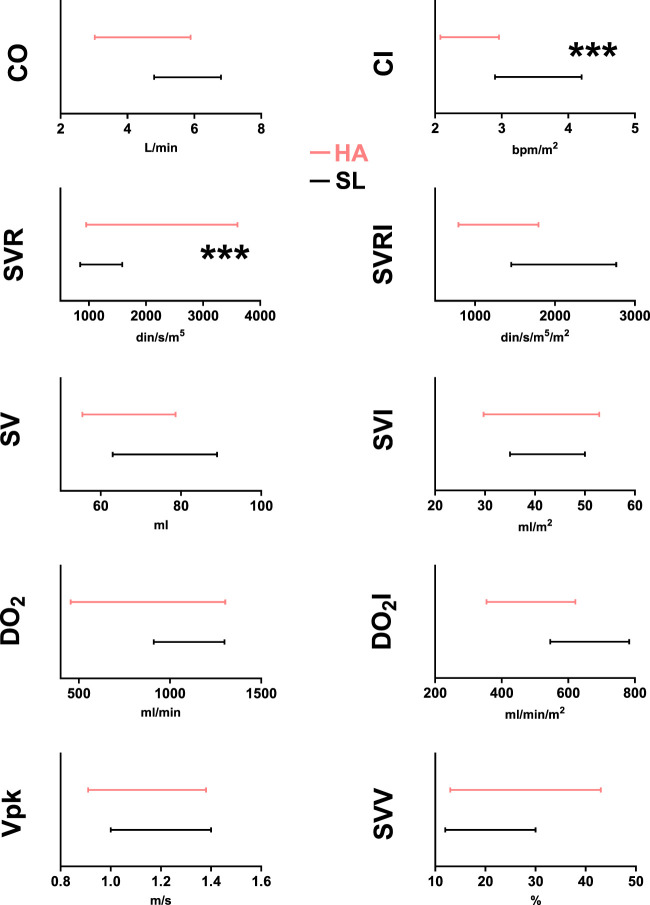
USCOM age-specific reference intervals for healthy female adult high-altitude (3,600 m–red lines) and sea-level (black lines) residents for CO (cardiac output), CI (cardiac index), SVR (systemic vascular resistance), SVRI (systemic vascular resistance index), SV (stroke volume), SVI (stroke volume index), DO_2_ (arterial oxygen delivery), DO_2_I (arterial oxygen delivery index), Vpk (peak ejection velocity), and SVV (stroke volume variation). ***: *p* < 0.001 HA vs. SL.

Taking advantage of USCOM’s provision of reference intervals for hemodynamic parameters among residents at sea level (Nguyen et al., 2010), we employed Moses extreme reaction tests to juxtapose them with data collected from high-altitude residents. While no differences were found in men (Figure 3 Table 5), among women, we found that the CI reference intervals of high-altitude residents are significantly higher than the corresponding intervals of sea-level residents, while the reference intervals for SVR are significantly larger (Figure 4 Table 5). Once more, the measurements unveiled by USCOM technology underscore significant disparities in hemodynamic parameters between permanent sea-level residents and those at high altitudes.

**TABLE 5:** Moses extreme reactions test results for the comparison between SL and HA reference intervals.

	Female	Male
**CO (L/min)**	G* = 5, *p* = 0.576	G* = 6, *p* = 0.682
**CI (L/min/m** ^**2**^ **)**	G* = 2, *p* < 0.0001	G* = 3, *p* = 0.318
**SVR (din/s/cm** ^**-5**^ **)**	G* = 2, *p* < 0.0001	G* = 12, *p* = 1.0
**SVRI (din/s/cm** ^**-5**^ **/m** ^**2**^ **)**	G* = 3, *p* = 0.318	G* = 11, *p* = 0.985
**SV (mL)**	G* = 11, *p* = 0.985	G* = 8, *p* = 0.848
**SVI (mL/m** ^**2**^ **)**	G* = 10, *p* = 0.955	G* = 12, *p* = 1.0
**DO**_**2**_ **(mL/min)**	G* = 6, *p* = 0.682	G* = 7, *p* = 0.773
**DO**_**2**_ **I (mL/min/m** ^**2**^ **)**	G* = 4, *p* = 0.455	G* = 4, *p* = 0.455
**Vpk (m/s)**	G* = 11, *p* = 0.985	G* = 12, *p* = 1.0
**SVV (%)**	G* = 11, *p* = 0.985	G* = 11, *p* = 0.985

CO (cardiac output), CI(cardiac index), SVR (systemic vascular resistance), SVRI (systemic vascular resistance index), SV (stroke volume), SVI (stroke volume index), DO_2_ (arterial oxygen delivery), DO_2_I (arterial oxygen delivery index), Vpk (peak ejection velocity), and SVV (stroke volume variation) are shown as mean ± SD.

## Discussion

In this study we performed a pilot investigation to validate the applicability of USCOM technology in elucidating the hemodynamic characteristics of healthy permanent residents living at high altitudes, (above 2,500 masl). The principal findings of our study indicate that data acquired through USCOM technology in this population: (i) show robust reproducibility, (ii) reveal sex-related hemodynamic profiles among high-altitude residents, and (iii) unveil substantial variations in hemodynamic parameters when compared to those of healthy sea-level residents.

Any validation process faces challenging considerations that lead to identifiable limitations. The limitations of our work, along with strategies to overcome them, are as follows; 1) Our research protocol requires the participation of healthy people to establish baseline measurements. For obvious reasons, convincing healthy people to undergo medical evaluations presents a significant obstacle. Our efforts to recruit individuals who met this study criterion spanned an arduous 8-month period, culminating in the enrollment of 28 subjects. Although this cohort may not be ideal for a large study, it is suitable for a pilot study, as demonstrated by the calculation of the required sample size (see material and methods section). 2) The USCOM equipment used in this investigation is the only device in existence in La Paz, Bolivia, and is presumably the exclusive apparatus operational solely at an altitude of 3,600 m. Consequently, the ambit of our study is inherently circumscribed within a single-center framework. 3) Transthoracic echocardiography stands as the preferred contemporary tool in clinical cardiology for the evaluation of hemodynamic parameters. Unfortunately, this technology is currently not available in Bolivian hospitals. In light of this limitation, USCOM technology emerges as a superior alternative, with several advantages over transthoracic echocardiography, including greater accuracy, validity, and precision. In particular, its inherent portability facilitates rapid and reliable measurements, making it accessible to physicians with appropriate training, avoiding the need for specialized cardiology expertise. Furthermore, as mentioned in the materials and methods section, all measurements were carried out by a single professional. This meticulous approach was adopted with the specific intent of mitigating potential measurement discrepancies that could arise from the involvement of multiple professionals in the data acquisition process. Furthermore, in support of the aforementioned, numerous previous studies corroborate the validity of the USCOM technique. In particular, the USCOM-1A device demonstrated accuracy compared to thermodilution in cardiac surgery patients within the intensive care unit (Tan et al., 2005). Similarly, good agreement was observed between the USCOM monitor and pulmonary artery catheter thermodilution in patients undergoing liver transplantation (Wong et al., 2008). Additionally, the USCOM monitor showed excellent interrater reliability and a short learning curve for stroke volume measurements in acute emergency admissions (Hodgson et al., 2016). Additionally, it has been shown that emergency physicians without ultrasound experience can be trained to use USCOM and reliably obtain cardiac output results noninvasively in conscious emergency department patients (Dey and Sprivulis, 2005). These collective findings reinforce the credibility and applicability of the USCOM device in various clinical settings.

Several earlier investigations have previously assessed hemodynamic parameters in Tibetan and Andean populations. These studies uniformly relied on pulmonary artery catheterization, acknowledged as the conventional technique for cardiac output measurement via thermodilution in humans. A comprehensive review of parameters facilitating adaptation to high-altitude hypoxia recently summarized the findings from these studies (Bell and Strohl, 2021). Notably, this synthesis revealed a broad range of resting cardiac index values (cardiac output normalized by body surface area, accounting for body size) among Tibetan and Andean highlanders. Their cardiac index values encompassed the medium to high end of the variation spectrum observed at lower altitudes. The substantial variability in these values can be attributed to the diverse altitude levels, experimental setups, and subjects employed in studies conducted between 1967 and 1995 (Bell and Strohl, 2021). Despite the valuable insights gained from these investigations, notable gaps remain in establishing basic reference values for these parameters. Furthermore, a widespread misconception persists in academia and medical research, which assumes that the physiological attributes of high-altitude populations can be directly extrapolated from characteristics delineated for sea-level inhabitants. Contrary to this notion, an unresolved gap remains in comprehensively quantifying the unique physiological parameters inherent to high-altitude environments. This accentuates the imperative necessity to reevaluate and redefine our understanding of the specific physiology of high-altitude inhabitants. Such a gap presents formidable challenges for clinicians in high-altitude locations, requiring accurate and contemporary data to inform critical decisions that can decisively affect patient survival.

The advent of the ultrasonic cardiac output monitor (USCOM) has emerged as a valuable solution to address this pressing clinical demand. The utilization of continuous wave Doppler ultrasound at elevated frequencies enables the non-invasive acquisition of real-time transcutaneous cardiac output measurements, extending its applicability to healthy individuals. In patients admitted in critical care, the USCOM monitor is accurate, rapid, safe, well-tolerated, non-invasive and cost-effective. The learning curve for skill acquisition is very gentle but it is necessary of “operator dependent” in the interpretation of results (Tan et al., 2005). Consequently, this study represents the pioneering application of USCOM technology to ascertain hemodynamic parameters in individuals who permanently reside at high altitudes. Our outcomes hold substantial clinical significance, as they reveal that USCOM measurements at these altitudes provide robust and highly reproducible readings. Furthermore, the data obtained have enabled us to elucidate significant sex-based disparities in hemodynamic performance, distinct from the influence of altitude of residence. These preliminary findings call for urgent development of a comprehensive study involving a larger and more diverse cohort. Such an effort would allow us to validate our initial observations and delve deeper into the physiological, cellular, and molecular alterations that underlie continuous exposure to a barometric pressure approximately one-third lower than that found at sea level.

In summary, our findings underscore the utility of ultrasonic monitoring technology for assessing hemodynamic parameters in healthy Andean individuals residing in high-altitude regions. Although these results are not extensible to permanent residents of other highlands, the high reproducibility of data obtained through the USCOM technique mitigates the substantial variability observed in previous hemodynamic studies. Additionally, our results reveal noteworthy disparities attributed to altitude and sex. These outcomes underscore the necessity for further research in high-altitude human populations to address knowledge gaps and enhance our comprehension of high-altitude physiology.

## Data Availability

The raw data supporting the conclusion of this article will be made available by the authors, without undue reservation.
